# Individual differences in experiential diversity shape event segmentation granularity

**DOI:** 10.1016/j.isci.2025.113134

**Published:** 2025-07-16

**Authors:** Carl J. Hodgetts, Samuel C. Berry, Mark Postans, Angharad N. Williams

**Affiliations:** 1 Department of Psychology, Royal Holloway, University of London, Egham, Surrey TW20 0EX, UK; 2 Communicable Disease Surveillance Centre, Public Health Wales, Number 2 Capital Quarter, Tyndall Street, Cardiff CF10 4BZ, UK; 3 Department of Psychology, Chaucer, Nottingham Trent University, 50 Shakespeare Street, Nottingham NG1 4FQ, UK; 4 Adaptive Memory Research Group, Max Planck Institute for Human Cognitive and Brain Sciences, Stephanstraße 1A, 04103 Leipzig, Germany

**Keywords:** Neuroscience, Social sciences

## Abstract

Parsing experience into meaningful events or units, known as event segmentation, may be critical for structuring episodic memory, planning, and navigating spatial and social environments. However, little is known about what factors shape inter-individual differences in event segmentation. Here, we show that individuals with greater variation in their daily social and spatial lives (experiential diversity) displayed more fine-grained event segmentation during a movie-viewing task. This relationship held after considering potential confounds, such as anxiety, loneliness, and socioeconomic factors, and was primarily driven by variation in social experiential diversity. Exploratory analyses revealed that the relationship between social experiential diversity and segmentation granularity was stronger in high-anxiety participants, suggesting heightened vigilance to fine-grained social-emotional cues during movie-viewing. These results support the view that event segmentation can occur proactively based on social and spatial environmental dynamics learned “in the wild” and provide a potential cognitive pathway through which isolation impacts cognitive health.

## Highlights

• Greater experiential diversity linked to finer-grained event segmentation
• Social, not spatial, experiential diversity drives this relationship
• Association stronger in high-anxiety individuals, suggesting increased vigilance
• Findings support proactive event segmentation based on real-world dynamics

## Graphical abstract

**Figure:**
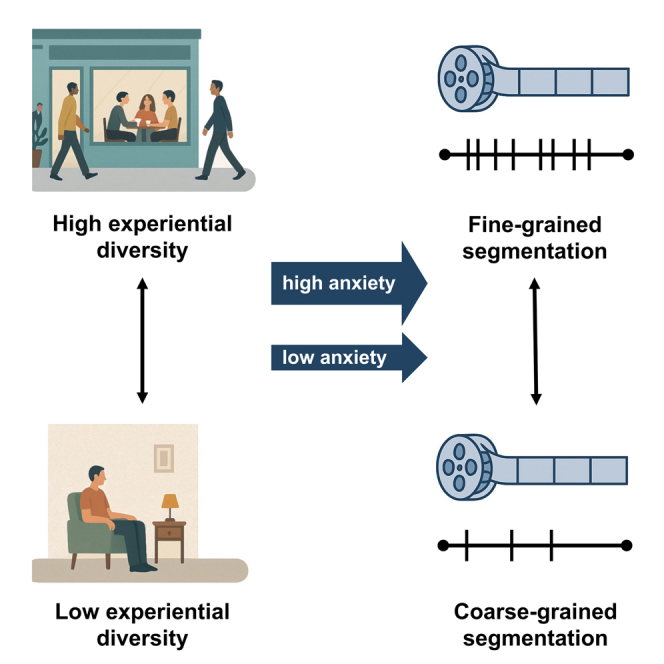

## Introduction

While the world provides a continuous stream of perceptual input, we perceive and remember our daily lives as a series of discrete events with distinct beginnings and endings. This process, known as event segmentation, is thought to rely on the detection of event boundaries.^1^^,^^2^ These boundaries can be estimated implicitly (e.g., by clustering fMRI activity into discrete states during continuous movie viewing)^3^^,^^4^ or measured directly by asking participants to press a button whenever they perceive one meaningful unit of activity to end and another to begin.^5^ While the precise neurocognitive mechanisms for determining event boundaries are still debated,^6^^,^^7^^,^^8^ there is growing evidence that event segmentation strongly influences several other aspects of cognition, including spatial navigation, long-term memory, and motor planning.^9^^,^^10^^,^^11^

Despite its potentially broad psychological relevance, there remains a limited understanding of how, and why, this ability differs across individuals. While prior work often emphasizes high agreement in where event boundaries are placed between individuals,^12^ there are nonetheless studies showing substantial inter-individual variation in the number of events segmented for a given stimulus^6^^,^^13^^,^^14^—a finding that follows from known individual differences in the granularity of event memories.^15^

As event representations are shaped and updated through interactions with the external world,^2^ one potential source of inter-individual variation could be the degree of situational change experienced in one’s physical and/or social environment. Indeed, people differ significantly in how much variability they encounter in their daily lives—referred to as “experiential diversity”.^16^^,^^17^

Evidence linking event processing to experiential diversity predominantly stems from studies in nonhuman species. For example, research on environmental and social enrichment in rodents suggest that experiential diversity is not only critical for psychological wellbeing (i.e., reducing stress) but promotes spatial-event memory alongside structural plasticity in brain regions associated with event processing in humans, such as the hippocampus and prefrontal cortex.^18^^,^^19^^,^^20^ In humans, markers of low social experiential diversity, such as social isolation, have been associated with memory impairment in older adults.^21^ Studies on spatial diversity indicate that individuals raised in areas with high street network entropy (i.e., more complex, less ordered environments) exhibit better navigation skills in adulthood^22^ (see also ref. 23). Similarly, studies involving both younger and older adults have shown that behavioral interventions that promote exploration within novel, large-scale environments lead to improvements in mnemonic discrimination, indicating a more fine-grained encoding of events.^23^^,^^24^ Collectively, these findings imply that exposure to rich, varied experiences may enhance event-related cognition and induce structural changes in brain regions implicated in event segmentation.^3^^,^^25^ However, it remains unknown whether real-world inter-individual differences in daily experiences are associated with the granularity of event segmentation itself.

An additional question relates to the expected direction of the relationship between experiential diversity and event segmentation. From the perspective of Event Segmentation Theory,^1^ low levels of real-world experiential diversity may increase event segmentation granularity due to more imprecise predictions about the world. Specifically, the lack of exposure to highly variable experiences may increase the probability that minor shifts in situational features and/or internal states cross an event horizon, and lead to the generation of a new event model. More recent data, however, suggests that higher experiential diversity could actually *increase* the granularity of event segmentation because regular exposure to complex physical and social environments could enhance sensitivity to changes in fine-grained situational features that comprise events (e.g., social and environmental cues)^26^ and their resulting internal states (e.g., goals and motivations).^17^^,^^27^ Understanding the relationship between experiential diversity and event segmentation, therefore, is not only relevant for understanding real-world psychological effects of low experiential diversity (such as those stemming from social isolation, loneliness, and poor physical or mental health), but is also important in the theoretical understanding of event segmentation as a cognitive process.

**Figure 1:**
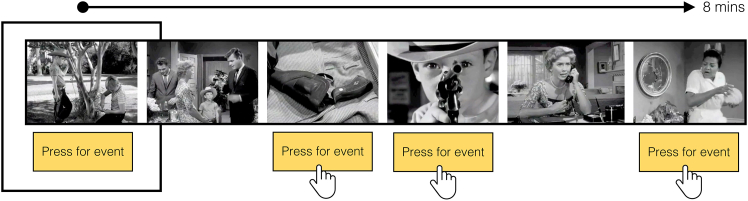
The event segmentation task The event segmentation task involved the presentation of an 8-min movie: Alfred Hitchcock’s black-and-white television drama *“Bang! You’re Dead”*. The movie was shown above an on-screen button, which participants pressed whenever they judged one event (i.e., “a meaningful unit of time”) to end and another to begin.

To address this, we examined the association between real-world experiential diversity and event segmentation granularity within a sample of 157 young healthy adults (see STAR Methods for participant details). Drawing on recent evidence,^8^^,^^17^ we hypothesized that higher experiential diversity would lead to more fine-grained event segmentation. For each participant, we assessed both social and spatial experiential diversity over the preceding 30-day period, allowing us to examine the differential contributions of each component.^28^ The social experiential diversity score was comprised of two sub-scales: one assessing the regularity and format of recent social interactions, and the other assessing social network size. The spatial experiential diversity questionnaire was designed to evaluate the complexity of each participant’s immediate domestic and local environment, including the number of rooms they spend their time in on a typical day, access to private outdoor space, and the frequency in which they explore their local neighborhood. Partial COVID-19 restrictions at the time of testing in the United Kingdom^29^—which restricted the size of social gatherings, access to work and education, and travel—also provided a unique opportunity to study the effects of reduced experiential diversity within a young, healthy adult sample. Participants also completed an event segmentation task in which they watched a short film—an abridged version of Alfred Hitchcock’s *“Bang! You’re Dead”* (see Figure 1)—and marked perceived event boundaries using an on-screen button^25^^,^^30^ (see STAR Methods). Given our focus on event segmentation granularity, our target measure was the number of perceived event boundaries^6^^,^^26^^,^^31^^,^^32^ reported by each participant during the movie stimulus.

## Results

### Experiential diversity is correlated with event segmentation granularity

**Figure 2:**
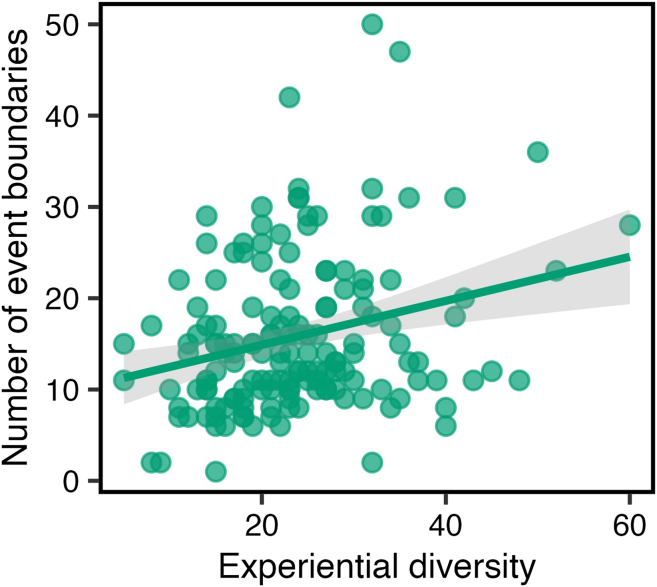
The relationship between total experiential diversity and event segmentation granularity Each data point reflects an individual participant and there are 157 data points shown. The shaded area represents the 95% confidence interval on the best-fitting regression line.

We predicted that individuals with greater experiential diversity would segment the movie stimulus into more fine-grained events, reflecting increased segmentation granularity (see introduction). To test this, we conducted a bivariate Pearson’s correlation between total experiential diversity (i.e., our composite of social and spatial subscales) and event segmentation granularity (Figure 2). Consistent with our prediction, we observed a significant positive correlation between experiential diversity and event segmentation frequency (*r*(155) = 0.27, *p* < 0.001).

### Controlling for socioeconomic factors, anxiety and loneliness

Given the observed relationship between experiential diversity and event segmentation, we next examined whether this effect remained when accounting for other potential confounding factors. In particular, lower experiential diversity—reflected, for example, in smaller social networks or limited access to varied spatial environments—may be associated with emotional and/or demographic variables that also influence event perception. For instance, mental health factors such as anxiety have been linked to memory alterations, including overgeneral memory (i.e., the tendency to recall events in a coarse, categorical manner), which may reflect the granularity of event segmentation (e.g., a bias toward coarser, less differentiated event boundaries).^33^^,^^34^ While the relationship between social network size and social isolation is complex, it is also possible that low social experiential diversity could contribute to subjective feelings of loneliness. However, these constructs are not interchangeable: individuals may report high levels of social contact but still experience loneliness, or conversely, maintain small social networks without feeling socially isolated.^35^ It is therefore important to distinguish between objective experiential diversity and subjective feelings of social disconnection. Further, some aspects of experiential diversity, such as time spent in green spaces or exposure to varied domestic environments, may be influenced by socioeconomic status (estimated here via household income and years of education).

To examine these potential influences, we conducted a multiple regression analysis with event segmentation granularity as the outcome, and experiential diversity, trait anxiety, and loneliness as predictors (see STAR Methods for details of each additional covariate). The significant relationship between experiential diversity and event segmentation granularity remained when controlling for both anxiety and loneliness (β = 0.25, *p* < 0.001). Further, neither anxiety (*p* = 0.10) nor loneliness (*p* = 0.19) independently contributed to inter-individual variation in event segmentation granularity.

We next examined whether this effect could be attributed to socioeconomic factors (see STAR Methods). To do so, household income and years of education were added as covariates in a separate regression model, conducted within a subset of participants for whom socioeconomic data were available (*N* = 146). As in the primary analysis, the relationship between experiential diversity and event segmentation remained significant when controlling for these factors (β = 0.23, *p* = 0.002), and neither socioeconomic variable was independently associated with experiential diversity (household income: r(144) = 0.14, *p* = 0.10; years of education: r(144) = −0.04, *p* = 0.60).

### The differential contribution of “social” and “spatial” experiential diversity

We next explored the relative contribution of social and spatial experiential diversity measures to event segmentation granularity. To determine whether these measures were sufficiently distinct to warrant statistical comparison, we first examined their inter-correlation. Social and spatial experiential diversity were positively correlated (r(155) = 0.29, *p* < 0.001), indicating some shared variance. However, the strength of this association was moderate and comparable to the effect observed between experiential diversity and event segmentation itself.

**Figure 3:**
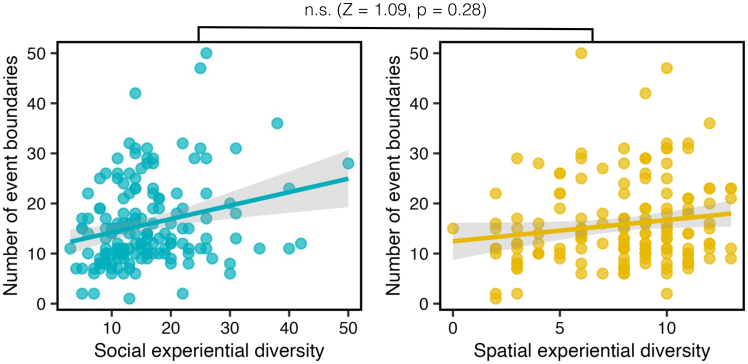
The relationship between social and spatial experiential diversity subscales and event segmentation granularity Statistical comparisons between social and spatial conditions are reported above the plots (Steiger Z-test). Each data point reflects an individual participant and there are 157 data points shown. The shaded area represents the 95% confidence interval on the best-fitting regression line.

When relating these sub-scales to event segmentation granularity, we found that only social experiential diversity was significantly associated with the number of event boundaries segmented (*social*: r(155) = 0.25, *p* = 0.001; *spatial*: r(155) = 0.15, *p* = 0.06; see Figure 3). While these correlations did not differ significantly from each other (Z = 1.09, *p* = 0.28), a multiple regression model incorporating both sub-scales showed that only social experiential diversity significantly accounted for variation in event boundary segmentations (β = 0.24, *p* = 0.006). This suggests that social experiential diversity is the main contributor to event segmentation granularity in this study.

### Social—but not spatial—experiential diversity is moderated by anxiety

**Figure 4:**
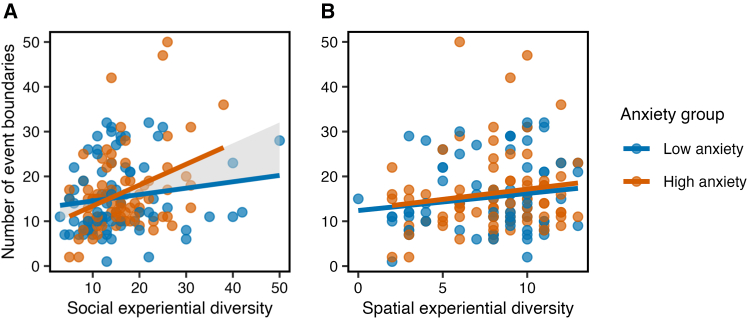
The relationship between (A) social and (B) spatial experiential diversity and event segmentation granularity as a function of anxiety A median split is used for visualization purposes, with 77 in the high anxiety group and 80 data points in the low anxiety group (157 data points in total). The shaded area represents the difference between the slopes.

Finally, we examined whether this relationship between experiential diversity and event segmentation granularity was moderated by individual differences in anxiety or loneliness. Having established a stronger relationship between *social* experiential diversity and segmentation granularity, we tested whether this specific effect varied as a function of these key mental health factors. A multiple regression including interaction terms revealed a significant interaction between social experiential diversity and trait anxiety (β = 0.07, *p* = 0.012). Specifically, the positive effect of social experiential diversity on segmentation granularity became stronger at higher levels of anxiety (see Figure 4A). In contrast, neither the interaction between spatial diversity and anxiety (Figure 4B), nor any interactions with loneliness, were significant (*p* values >0.36). These findings suggest that anxiety may enhance sensitivity to social cues during event perception, particularly in individuals with more diverse social experiences (see discussion).

## Discussion

In this study, we investigated the association between individual differences in real-world experiential diversity (e.g., the frequency and quality of social interactions, and the capacity to explore the immediate spatial environment) and variation in event segmentation granularity. Our results yielded several key findings. First, we found a significant relationship between experiential diversity and the number of event boundaries perceived during a movie-viewing task, such that participants who had recent short-term exposure to a wider range of social and spatial experiences provided more fine-grained segmentations of events as they unfolded. Second, this effect remained significant when controlling for potential confounding factors, including trait anxiety, loneliness, and socioeconomic status. Third, when comparing social and spatial experiential diversity, we found that social experience accounted for a greater proportion of variation in event perception compared to spatial experiences, although the difference between these correlations was not statistically significant. Finally, we observed that anxiety moderated the effect of social experiential diversity, such that the association with segmentation granularity was stronger among individuals with higher levels of trait anxiety.

The observed relationship between the complexity and diversity of everyday experience and event segmentation granularity aligns with the view that event segmentation can be actively shaped by prior knowledge and expectations about environmental dynamics—rather than solely reflecting prediction error (see e.g., ref.^8^ for recent empirical demonstration in the context of temporal order memory). For example, Event Segmentation Theory may predict that low experiential diversity (characterized by fewer major shifts in an individual’s everyday environment) would lower the threshold for triggering a new event model via prediction error, resulting in *increased* sensitivity to event boundaries.^1^^,^^36^ We propose, however, that rather than reacting to prediction errors, event segmentation in individuals with high experiential diversity may segment in a more proactive manner, unitizing experiences and internal states^17^^,^^27^ based on a richer, more fine-grained understanding of the environment’s temporal dynamics and structure.^7^^,^^8^

This interpretation aligns, in part, with research on expertise and event perception. For example, basketball experts generate more event boundaries compared to novices when watching basketball games, particularly when attending to fine-grained events^26^ (but see ref. 37). Thus, it may be that participants’ boundaries aligned at the coarse-level in the current study (i.e., for salient scene changes) but that individuals with higher experiential diversity had increased sensitivity to finer-scale spatial and/or social features of the narrative.^9^ This interpretation may also feasibly extend to internal features, such as emotional states, goals, and motivations.^2^^,^^38^ Indeed, recent work has shown that individuals exposed to more varied social contexts display greater emotional granularity.^17^^,^^27^ Thus, these effects may reflect a combined sensitivity to both external situational cues and internal states (with the latter more challenging to detect in our experimental design).

Because our online experimental setup did not capture the precise timing of boundary placements, we were not able to determine which specific situational features drove segmentation. However, future studies could examine whether boundary placement is tied to specific situational features (e.g., social information), and whether this is related to specific aspects of experiential diversity. Prior work has shown that lonely individuals show enhanced vigilance to negative social information,^39^ suggesting that reduced social experiential diversity may increase sensitivity to certain event boundaries. However, loneliness did not significantly correlate with event segmentation in the current study, and—notably—the relationship between experiential diversity and segmentation ability held when controlling for loneliness in our analyses. This is important as it implies that reductions in social contact alone may be sufficient to impose real-world psychological changes, independent of subjective feelings of loneliness.

Experiential diversity may also influence event segmentation by altering the functional integrity of a core brain network linked to event cognition.^3^^,^^30^ Studies in rodents have reported significant declines in spatial memory when compared to those housed in groups, and this is associated with a host of structural changes in the hippocampus and prefrontal cortex (including demyelination and neuroinflammation).^40^^,^^41^ Further, changes in these markers may also be mitigated by later exposure to enriched spatial environments,^42^ which may even reverse the effects of isolation on hippocampal plasticity.^43^ Human studies similarly support the influence of experiential diversity on this brain network (see e.g., ref. 44). For instance, sudden and prolonged isolation (i.e., in polar expeditioners) has been linked to lower hippocampal volumes relative to controls.^45^ Additionally, engaging in complex exploratory behavior (e.g., in the local neighborhood) has been linked to positive affect, and increased hippocampal-striatal connectivity.^16^ Together, this cross-species evidence underscores the close relationship between everyday social and spatial experiences and the brain systems supporting event processing.^28^^,^^46^^,^^47^^,^^48^

It is important to note that this study was conducted as COVID-19 restrictions were being gradually lifted in the United Kingdom, meaning that many children and adults had undergone a prolonged and acute phase of isolation, with restrictions on social interaction, work, education, and travel. As such, it is possible that social and spatial experiences carried greater psychological salience than they would under typical circumstances.^49^ This highlights the importance of replicating these findings in less restrictive contexts and across different populations. Moreover, because of this broader context, individuals who scored low on our experiential diversity measure (which captured the 30 days prior to testing) may have experienced an even more sustained period of limited diversity. Given the well-established relationship between stress and cognition,^50^ the observed effects may be amplified in this sample due to prolonged exposure to environmental stressors. Animal studies have shown that stress significantly disrupts global remapping in hippocampal place cells—specifically, impeding the ability to efficiently shift spatial representations across contexts.^51^ It is therefore possible that individuals with low experiential diversity demonstrated stress-related reductions in representational flexibility when faced with changes in spatial and/or social environmental features, as reflected in the perception of fewer event boundaries.

While our primary results held when controlling for anxiety, additional exploratory analyses revealed that anxiety also moderated the relationship between social experiential diversity and segmentation granularity. Specifically, the positive association between social experiential diversity and segmentation frequency was enhanced in individuals with higher trait anxiety. This finding suggests that, under certain conditions, anxiety may enhance sensitivity to social/emotional features during event perception—perhaps by enhancing vigilance or attention to salient social-emotional transitions.^52^^,^^53^

Overall, this study demonstrates a link between experiential diversity and event segmentation granularity, offering novel insights into how real-world variation in social and environmental connectedness may shape cognition. Understanding these relationships has important implications for interventions aimed at enhancing cognitive health through increased experiential diversity. Future work should focus on better understanding how experiential diversity shapes encoding of different forms of event-related information (e.g., social vs. non-social information) across the lifespan, and how this relates to “downstream” aspects of cognition, including episodic memory and navigation.

### Limitations of the study

While results indicate greater sensitivity to fine-grained social-emotional cues, our task design did not allow us to determine when event boundary responses occurred. Future studies will be needed to determine the specific situational and/or emotional features that drive segmentation granularity—for example, by explicitly directing participants to identify particular types of boundaries^9^ or by relating timestamped boundary presses to independent ratings of movie content. It is also possible that our measure of social experiential diversity captured variation in spatial experiences, as social connectedness may lead to more exploratory behavior.^54^ In addition, our study was conducted during partial COVID-19 restrictions, which may have increased the psychological salience of social and spatial experiences. Nonetheless, these results offer insight into cognitive function under real-world conditions of environmental constraint and unpredictability—contexts that are highly relevant from a public health perspective.

## Resource availability

### Lead contact

Further information and requests for resources should be directed to and will be fulfilled by the lead contact, Carl J. Hodgetts (carl.hodgetts@rhul.ac.uk).

### Materials availability

No new materials were generated in this study.

### Data and code availability

• All de-identified data supporting the findings of this study are available on the Open Science Framework (https://osf.io/2q4wr/).
• All code for the analyses and figures presented in the paper are available on the Open Science Framework (https://osf.io/2q4wr/).
• Any additional information is available from the lead contact upon request.

## Acknowledgments

C.J.H. and S.C.B. were supported by the 10.13039/501100000268Biotechnology and Biological Sciences Research Council (BBSRC) [BB/V010549/1]. For the purpose of open access, the author has applied a Creative Commons Attribution (CC BY) license to any Author Accepted Manuscript version arising. We would also like to thank Saloni Krishnan, Nura Sidarus, and Andrew Lawrence for their valuable discussion.

## Author contributions

C.J.H.: Conceptualization, investigation, formal analysis, data curation, project administration, visualization, writing – original draft, writing – reviewing and editing. S.C.B.: Conceptualization, writing – reviewing and editing. M.P.: Conceptualization, investigation, formal analysis, writing – reviewing and editing; A.N.W.: Conceptualization, investigation, formal analysis, writing – reviewing and editing.

## Declaration of interests

The authors declare no conflicts of interest.

## STAR★Methods

### Key resources table

**Table:** 

REAGENT or RESOURCE	SOURCE	IDENTIFIER
**Deposited data**		

De-identified data and analysis code	Center for Open Science https://www.cos.io/	https://osf.io/2q4wr/

**Software and algorithms**		

R 4.4.3	The R Project for Statistical Computing	https://www.r-project.org/
Qualtrics (2023)	Qualtrics, Provo, UT, USA	https://www.qualtrics.com/

### Experimental model and study participant details

Young and healthy human participants were recruited from the participant recruitment platform Prolific (https://prolific.ac) and reimbursed for their participation at a rate of £8.49/hr. Participants were between 18-35 years of age, UK residents, fluent English speakers, and had no current or previous neurological or psychiatric conditions. Based on an *a priori* power analysis, we aimed to collect a total of 153 complete datasets in a sample of young adult human participants (which would provide 80% power to detect a one-tailed, medium effect size of r = 0.2). A total of 177 participants engaged with the study on Prolific, and 157 provided complete data for our key variables of interest (mean age = 26.4; SD = 5.4; range = 18-35). Participants’ gender was recorded using ‘female’ and ‘male’ options, with “Another gender not listed here” option where they could self-identify their gender. Ninety participants reported being female and 65 reported being male. One participant reported their gender as nonbinary, and one as trans. Ethnicity was not collected in this study. This study was approved by the Royal Holloway Department of Psychology Research Ethics Committee (ethical approval reference 2171).

### Method details

#### Event segmentation task

Participants from Prolific were directed to Qualtrics (Provo, UT; www.qualtrics.com) to complete the experiment. The paradigm was optimised for computers, phones, and tablets. For the task, participants watched an abridged version of Alfred Hitchcock’s black-and-white television drama *“Bang! You’re Dead”* (for applications of this movie stimulus in functional MRI studies, see ref.^25^^,^^30^). This film was chosen as it is highly suspenseful, but also involves dynamic social interactions across multiple scenes and locations and is therefore ideal to examine the cognitive-perceptual consequences of social and spatial experiential diversity. This contrasts with many other studies of event segmentation, which have used videos depicting a single agent performing action sequences or daily activities.^5^^,^^55^ Importantly, no participant reported seeing this film before, meaning that perceived events were not driven by prior knowledge of the film itself. During the movie, participants were required to press an on-screen button (using their mouse or phone/tablet touchscreen) whenever they judged one event to end and another to begin (Figure 1). They were instructed that there were “no right answers in this task” and to just respond in a way that feels natural to them. The main measure of event segmentation was the total number of perceived event boundaries per participant (i.e., the total number of button presses during the movie).^6^^,^^26^^,^^31^^,^^32^

#### Experiential diversity measures

The experiential diversity measure was designed to capture the diversity of each participant’s ‘social’ and ‘spatial’ experiences over the preceding 30 days. The social experiential diversity score was comprised of two sub-scales. The first included several questions assessing the regularity and format of recent social interactions (see Table S1). The second scale was a measure of social network size,^56^^,^^57^ whereby participants provided the initials of every individual they had had meaningfully contacted over the last 30 days. This questionnaire is thought to probe the second layer of an individual’s social network (the ‘sympathy group’),^58^ and the 30-day limit is thought to maximise variation across individuals while also minimising the time and effort required to complete the questionnaire. The social experiential diversity score was a composite of these sub-scales, and participants’ scores ranged from 3-50 (mean = 15.9, SD = 8). The spatial experiential diversity questionnaire was designed to assess the complexity of each participant’s immediate domestic and local environment, including the number of rooms they spend their time in on a typical day, access to private outdoor space, and the frequency in which they explore their local neighbourhood (see Table S2). Scores on this measure could range from 0 to 16 (mean = 7.9, SD = 3, range in sample = 0-13). Scores on the social and spatial measures were combined into an overall experiential diversity score (mean = 23.8, SD = 9.3, range = 5-60).

#### Additional measures

In addition to our main measures above, we also collected data on several covariates of interest. The first of these was The Campaign to End Loneliness Measurement Tool,^59^ which is a 3-item tool to assess subjective feelings of loneliness. The three items on this tool are: *“1. I am content with my friendships and relationships”; “2. I have enough people I feel comfortable asking for help at any time”; “3. My relationships are as satisfying as I would want them to be”*. Participants could respond to each statement on a 5-point scale: ‘Strongly disagree’ (coded as 4 points); ‘Disagree’ (coded as 3 points); ‘Neutral’ (coded as 2 points); ‘Agree’ (coded as 1 point) and ‘Strongly agree’ (coded as 0 points). This coding scheme produces scores ranging from 0-12, where higher scores indicate higher levels of subjective loneliness.

To control for general feelings of anxiety (see results), participants also completed the trait component of the State-Trait Anxiety Inventory (STAI-T).^60^ The STAI-T contains 20 items assessing the frequency of anxiety generally felt by participants, and participants respond on a four-point scale from ‘Almost Never’ to ‘Almost Always’. Scores can range from 20-80 with high scores indicating high levels of general anxiety. Further, we estimated current socioeconomic status using two indicators: 1) educational attainment, coded from 0 (“No academic qualifications”) to 3 (“Postgraduate degree”); and 2) current household income, coded on a 9-point ordinal scale ranging from 1 (“< £5,200”) to 9 (“> £78,000”). Responses marked as “Prefer not to say” or “Other” were treated as missing, which resulted in a sample size of N = 146 for analyses involving these measures. As the two variables were on different scales (see above), they were standardised (z-scored) before being included in our regression models, allowing for equal weighting. These standardised variables were included as covariates in the regression analyses to control for the influence of socioeconomic status on the relationship between experiential diversity and event segmentation granularity.

### Quantification and statistical analysis

All statistical analyses were conducted using R (version 4.0.2; R Core Team, 2019) in RStudio (version 1.4.1106; RStudio Team, 2021) and are reported in the results section. For descriptives, means and standard deviations are reported, and the 95% confidence interval is shown for the best-fitting regression line on all scatterplots. Key correlational analyses (e.g., between experiential diversity and event segmentation granularity) were conducted using two-tailed bivariate correlations, with the contribution of potential covariates explored using partial correlations and multiple regression analyses. Partial correlations were carried out using the ‘ppcor’^61^ package in R. Comparisons between correlation coefficients were performed using Steiger Z-tests (Steiger, 1980) within the ‘cocor’ package in R.^62^ Multiple regression analyses were used to test the robustness of the main association while adjusting for potential confounding variables, including trait anxiety, loneliness, and socioeconomic status (see ‘additional measures’, above). Further, we conducted follow-up regression models to explore whether reported relationships between experiential diversity and event segmentation granularity varied as a function of anxiety or loneliness. Model fit was evaluated using beta coefficients, and variance inflation factors (VIFs) were computed using the car package to assess multicollinearity in regression models (which flagged no issues across analyses). Data were visualised using ggplot2. A total of 157 participants were included in all analyses, except those involving socioeconomic status measures. Eleven participants (N = 11) chose not to provide socioeconomic information, resulting in a sample size of 146 (N = 146) for these analyses (see method details).
