# Supplementary material for: Individual differences in experiential diversity shape event segmentation granularity
Source: iScience. 2025 Jul 16;28(8):113134. doi: 10.1016/j.isci.2025.113134 (PMC12432452; doi:10.1016/j.isci.2025.113134)
Supplement: Document S1. Tables S1 and S2 [file mmc1.pdf]

iScience, Volume *28*

Supplemental information

Individual differences in experiential diversity shape event segmentation granularity

Carl J. Hodgetts, Samuel C. Berry, Mark Postans, and Angharad N. Williams

Table S1

Table S1. *Social experiences questionnaire*

Considering your social interactions over the last month (i.e., 30 days), please indicate whether the following statements apply to you or not.<br><br>In the last month....		
**Question**	**Option 1**	**Option 2**
1. I have lived alone	No	Yes
2. I have had regular (i.e., weekly) face-to-face contact with close family or friends	No	Yes
3. I have had regular (i.e., weekly) voice or video call contact with close family or friends?	No	Yes
4. I have regularly attended a workplace, community group, sports club, and/or volunteering scheme with other individuals	No	Yes
5. I have regularly used messaging apps and social media to stay in touch with close family or friends	No	Yes
6. I have had regular contact with other individuals in my local community who are not primarily my friends of family (e.g., carer, delivery person, shopkeeper, neighbour, plumber, etc)	No	Yes
**Scoring**<br>Each item is scored 1 point for the 'Yes' response, except for Item 1, which is reverse coded (1 point = 'No'). Scores are between 0 and 6.		

Note. The social experiences questionnaire is designed to probe the regularity and format of recent social interactions. To derive an overall measure of social experiential diversity, this scale was combined with a measure of social network size, in which participants provided the initials of every individual they had had meaningfully contacted over the last 30 days (see main text).

Table S2

Table S2. *Spatial experiences questionnaire*

Question	Response options				
On a typical day within the last month (30 days), how many rooms did you spend your time in?	1	2-4	5-7	8+	
Do you have access to a private outdoor space?	No	Yes			
On a typical week in last month, how often did you leave your home (e.g., for exercise, shopping, errands)?	0	1	2-4	5-7	7+
Within the last month (30 days), how often have you visited recreational green space (e.g., woodland, parkland, forest)?	Never	At least once a month	At least once a fortnight	At least once a week	Every day
In the last month (30 days), how often have you taken a leisurely wander or stroll without any clear aim or goal?	Never	At least once a month	At least once a fortnight	At least once a week	Every day
**Score allocated**	0	1	2	3	4

Note. The spatial experiential diversity questionnaire is designed to assess the complexity of each participant's immediate domestic and local environment, including the number of rooms they spend their time in on a typical day, access to private outdoor space, and the frequency in which they explore their local neighbourhood (see main text).
